# Diagnostic accuracy of SARS-CoV-2 Panbio™ rapid antigen diagnostic tests in a 4,440-case clinical follow-up

**DOI:** 10.3389/fmed.2022.908127

**Published:** 2022-08-02

**Authors:** Ágoston Hamar, Kristóf Filipánits, Alex Váradi, Rita Váradi-Rácz, Henrietta Orsolya Gellén, Krisztina Futács, Péter Urbán, Gabor L. Kovacs, Katalin Gombos

**Affiliations:** ^1^Department of Laboratory Medicine, Medical School, University of Pécs, Pécs, Hungary; ^2^Institute for Translational Medicine, Medical School, University of Pécs, Pécs, Hungary; ^3^Genomics and Bioinformatics Core Facility, János Szentágothai Research Centre, University of Pécs, Pécs, Hungary; ^4^Molecular Biology Cluster, János Szentágothai Research Centre, University of Pécs, Pécs, Hungary

**Keywords:** COVID-19, SARS-CoV-2, rapid antigen testing, Panbio, RT-qPCR, diagnostic accuracy

## Abstract

Severe Acute Respiratory Syndrome Coronavirus-2 (SARS-CoV-2) Rapid Antigen Detection Testing (RADT) has been subjected to several evaluations in reference to diagnostic accuracy, ranging from small scale up to large population studies including nation-wide community-based studies. All confirmed the diagnostic accuracy of the tests which were strongly dependent upon the infection's population prevalence. In our retrospective study, parallel SARS-CoV-2 Panbio™ RADT assay, including real-time reverse transcription quantitative polymerase chain reaction (RT-qPCR) tests, were aimed to evaluate diagnostic performance regarding the rapid antigen diagnostic testing. Out of 4,440 paired tests, 609 samples tested positive using RT-qPCR, resulting in a prevalence of 13.7%. Panbio detected 251 (5.7%) positive tested samples. Overall sensitivity was 41.2% (95% CI 37.4–45.2%) and overall specificity was 99.7% (95% CI 99.4–99.8%). Positive predictive value (PPV) was 95.1% (95% CI 91.8–97.1%) and the negative predictive value (NPV) was 91.4% (95% CI 90.5–92.2%). RADT sensitivity increased with stratification in reference to the results according to PCR Cycle threshold (Ct) and presence of the symptoms considerably influenced PPV and NPV. Sensitivity in the group of Ct values ≤ 20 was 91.2%, 68.6% within the Ct range of 20–25, 47.9% in the group of Ct values between 25 and 30, and 12.6% in the group of Ct values between 30 and 35. A follow-up of the positive cases aligned with RT-qPCR testing and comparison of the general population enrolled in the testing in which the fatal cases occurred enabled us to estimate real clinical diagnostic performance regarding the SARS-CoV-2 Panbio RADT. Based upon our results, we recommend the SARS-CoV-2 Panbio RADT tests be carried out as the primary test, without parallel PCR testing, only among high population prevalence rates of the infection and to be used for symptomatic individuals with average or low severe disease developmental risk. In the case of high risk regarding the development of severe infection complications, a parallel SARS-CoV-2 RT-qPCR is needed to be carried out to attain proper diagnostic accuracy and avoid delaying appropriate medical care.

## Introduction

440.8 million cases of confirmed severe acute respiratory syndrome coronavirus 2 (SARS-CoV-2) infections and 5.6 million deaths from the coronavirus disease 2019 (COVID-19) have been, thus far, reported worldwide to the World Health Organization (WHO) (1). Public health and clinical measures used to control the pandemic and reduce disease burden require rapid tools for identification of the pathogen. Today, there are more than 400 different antigen-based immunoassay tests commercially available to detect SARS-CoV-2 on the market (2). The test is also widely available for clinical and community use throughout European countries, including Hungary. As the virus acquires increasing transmissibility in the various epidemic waves caused by the evolutionary selection of higher receptor specific and immune system escaping SARS-CoV-2 viral lineages, higher proportions of infected population enter the health care system (3, 4). Although RT-qPCR is the gold-standard technique considered to be the ultimate reference laboratory method to diagnose the presence of SARS-CoV-2, rapid antigen detection testing (RADT) is gaining traction since its rapid turnaround, cost effectiveness, utility at point-of care and reduced reliance on laboratory infrastructure (5). Several studies have shown a wide range of sensitivities associated with SARS-CoV-2 RADTs which is very dependent upon the viral load. Sensitivity can range from 95%, when a high concentration of viral capsid antigens are present in the sample, to 10–30% when the viral load is low (6, 7). In most clinical cases, SARS-CoV-2 RADT results needed to be confirmed by qPCR. Additionally, confirmatory PCR testing is recommended in the guidelines of several public health organizations (8–10). In health care systems, the goal of the test strategy is maximization of case detection while minimization of the unnecessary repetitive testing causing low productivity is of primary importance. In our retrospective study, we aimed to compare the SARS-CoV-2 Panbio RAD test performance with SARS-CoV-2 RT-qPCR to define the diagnostic accuracy of rapid testing in a clinical follow-up.

## Materials and methods

### Study details

Our retrospective study analyzed data gathered in the time spanning from 21 January 2021 through 30 April 2021. A total number of 5,136 parallel Panbio RADT and RT-qPCR samples were included from all departments of the Clinical Center, University of Pécs, Hungary. During the study protocol, all patients tested with Panbio RADT were tested in parallel by for SARS-CoV-2 RT-qPCR. Inclusion criteria were the presence of a SARS-CoV-2 RAD Panbio test result combined with an RT-qPCR test result, both performed within 24 h. Presence of symptoms were documented at the time of the parallel testing. Patients who were identified as positive cases with SARS-CoV-2 RT-qPCR were followed up by repeated PCR testing until their first negative PCR test.

Panbio RAD tests were performed and evaluated by trained health care professionals. The diagnostic PCR tests were carried out in the Department of Laboratory Medicine in full accordance to a protocol accredited by the National Accreditation Authority (NAH-9/0008/2021, L7/6 MLMB 06 2020.4-1).

### Sample collection

Two nasopharyngeal swabs were taken from patients by trained nurses and/or medical doctors in the specialized units of the Pécs University Clinical Center. The Panbio RAD test was performed immediately after obtaining the first nasopharyngeal swab in full compliance to the manufacturer's instructions. The second nasopharyngeal swab was washed into the sample collection tube containing virus transport medium (CE certified, Biolabs Ltd., Hungary). Swabs were broken at the groove and the remaining portion of the swabs were removed and safely discarded. Sample collection tubes were individually wrapped in sterile double-walled plastic bags and transferred to the laboratory at 4° C for nucleic acid extraction. Patients and Guardians accompanying underage patients were also tested. Healthcare employees were tested after having been in close contact to a confirmed SARS-CoV-2 positive individual, or if they exhibited respiratory symptoms. Individuals were considered symptomatic according to COVID-19 case definition of the ECDC (e.g., loss of smell or taste, sore throat, fever, dry cough, myalgia, etc.) (11, 12).

### Nucleic acid extraction and reverse-transcription quantitative real time PCR (RT-qPCR)

Nucleic acid was extracted from 200 μl specimen, whether manually or with the MagNaPure 96 automated nucleic acid extraction system (Roche, Mannheim, Germany). Automated extraction was optimized using the MagNA Pure 96 DNA and Viral NA SV Kit (Cat No. 654358800, Roche) in full accordance to the manufacturer's protocol. Additionally, during manual nucleic acid extraction, the HighPure RNA isolation kit (Cat No. 11858882001, Roche) was used in full compliance to the manufacturer's recommended protocol. Both automated and manual nucleic acid extraction procedures included LightMix Modular EAV RNA extraction control (Cat No. 61090996, TIB Molbiol) to verify extraction and reverse transcription. Five microliter of the extracted RNA was used for the rRT-qPCR analysis to detect the presence of SARS-CoV-2 RNA targeting three regions of the positive-sense single stranded viral genome: conserved fragments of the sequences encoding the envelope protein (E-gene), nucleocapsid protein (N-gene) and RNA dependent RNA polymerase (RdRP- gene). LightMix Modular SARS-CoV (COVID19) E-gene (Cat No. 53077696), LightMix Modular SARS-CoV (COVID19) N-gene (Cat No. 53077596) and LightMix Modular SARS-CoV (COVID19) RdRP (Cat No. 53077796) were used combined with the LightMix Modular EAV RNA extraction control 610 for simultaneous PCR target and extraction control detection. PCR Master mixes were prepared containing 0.5 μl of target specific primer and probe mix, 0.5 μl extraction control target specific primer and probe mix, 4 μl of Real Time ready Virus Master reaction buffer and 0.4 μl Real Time ready Virus Master RT enzyme (Cat No. 05992877001, Roche), 10.4 μl PCR grade water and 5 μl of the RNA sample. In consideration regarding the negative controls, we prepared a master mix in which template RNA was substituted with PCR grade water. PCR amplification was carried out using Cobas Z 480 PCR systems with the following cycling conditions (reverse transcription 1 cycle: 55° C for 5 min; enzyme activation 1 cycle: 95° C for 5 min; amplification 45 cycles {95° C for 5 s, 60° C for 15 s, and 72° C for 15 s}, Results were analyzed, and fluorescence data were evaluated using Exor 4.0 software. Cycle threshold (Ct) values were calculated with Exor 4.0. The kit manufacturer's instruction and the FIND evaluation guideline advised to define the cut-off 1–2 cycles higher than observed Cp value for 10 copies. During the kit verification run, it measured 33.14 on average lowest 10/10 dilution, which resulted in our cut-off to be fixed at Ct 35.0. To control PCR efficiency and potential pipetting errors, standard curves were generated on quantitative real-time PCR based on the dilution series of the positive controls provided with the LightMix Modular E, N and RdRP kits, which we previously quantitatively analyzed using the droplet digital PCR system (BioRad QX200 ddPCR platform and BioRad ddPCR Expert Design Assay: 2019-nCoV CDC ddPCR Triplex Probe Assay). PCR efficiency was calculated based on the present standard dilutions in the run. PCR results were accepted on the plate in the case of standard dilutions performed ≤ 0.2 log10 difference from the corresponding dilutions of the standard curves in case of each target gene (E, N, and RdRP). Plates containing low or high Ct outliers according to the in-run standard dilutions were repeated. In the following statistical analysis, we included the Ct results of the tested samples according to the SARS-CoV-2 E-gene.

### Statistics and data management

The test results and demographic data were originally documented in the local hospital information system (e-MedSolution, T-Systems, Hungary). Our extracted data was registered using Excel 2015 (Microsoft, Redmond, WA, USA). The final database includes an anonymized ID from both name and insurance number. It also contains information referencing gender, age, time and place (department) of test, RAD and PCR test result, Ct value, presence of symptoms and number of days until a negative PCR test in the event of a previous positive PCR test and mortality.

All statistical calculations were performed in R Statistics version 4.0.3 (R Foundation for Statistical Computing, Vienna, Austria). In regards to descriptive statistics, we reported frequencies and percentages for categorical variables, or mean with standard deviation (SD), median with interquartile range (IQR) and minimum and maximum values for continuous variables. In tables of descriptive statistic for each variable, an overall summary statistic of our sampled population is represented, and the statistics of the compared groups are shown in two separate columns and corresponding *p*-value for the applied statistical test is given. The *p*-values depicted in a row consistently refer to a comparison between the two columns highlighted in bold face. Negative PCR test results are shown as “not available” (NA) in the Ct value related tables. Chi-square test or Fisher's exact test have been used to investigate independence between two categorical variables. In the case of continuous variables, we used Wilcoxon rank sum test to assess the difference between medians of the two groups. Confidence interval calculations were executed using the Wilson/Brown method (13). A *p*-value <0.05 was defined as a two-tailed level of significance.

### Ethical issues

In this study, data were collected retrospectively and analyzed compliant to all ethical requirements. Ethical approval was granted by the Regional Committee for the Research Ethics at the University of Pécs Clinical Center and assigned reference number 8668-PTE 2021.

## Results

A total number of 5,136 samples tested parallel for SARS-CoV-2 Panbio RADT and SARS-CoV-2 RT-qPCR were collected from 4,440 individuals who were admitted in the clinical departments of the University of Pécs, Clinical Center (Table 1).

**Table 1:** Sample percentages per department.

**Emergency medicine**	**48.1%**
**Pediatrics**	**22.5%**
**Internal medicine (multiple wards)**	**10.1%**
**Obstetrics and gynecology**	**7.4%**
**Neurology and neurosurgery**	**3.7%**
**Other**	**8.3%**

### Demographics and clinical symptoms correlated with test results

The tested individuals were between 0 and 101 years old (median age: 53 years, IQR 30–72 years). The female/male ratio was 57.2%/42.8%. The median Ct values were significantly lower in the symptomatic group when compared with the asymptomatic group (28.2 vs. 35.0, respectively *p* < 0.001). Table 2 represents the Panbio RADT and RT-qPCR test results and Ct values in asymptomatic and symptomatic individuals. There were 1,249 cases (24.3%) in the symptomatic group. Two hundred and fifty-six out of 279 (91.8%) positive Panbio tests were taken from symptomatic patients.

**Table 2:** Presence of symptoms.

**Characteristic**	***N***	**Overall,** ***N* = 5,136**	**Symptomatic**		***p*-value**
			**No, *N* = 3,887**	**Yes, *N* = 1,249**	
**Panbio result**	5,136 (100.0%)				<0.001^a^
Negative		4,857 (94.6%)	3,864 (99.4%)	993 (79.5%)	
Positive		279 (5.4%)	23 (0.6%)	256 (19.5%)	
**PCR result**	5,136 (100%)				<0.001^a^
Negative		4,401 (85.7%)	3,661 (94.2%)	740 (59.3%)	
Positive		735 (14.3%)	226 (5.8%)	509 (40.7%)	
**Cycle threshold**	735 (14.3%)				
Mean (SD)		29.2 (5.7)	32.6 (3.6)	27.7 (5.9)	
Median (IQR)		30.6 (25.1, 35.0)	35.0 (31.1, 35.0)	28.2 (23.2, 32.9)	<0.001^b^
Minimum; Maximum		11.0; 35.0	20.8; 35.0	11.0; 35.0	
NA		4,401	3,661	740	

a *Pearson's Chi-squared test.*

b *Wilcoxon rank sum test*.

### Analytic performance of the antigen testing

We calculated the analytic performance in reference to the overall number of paired SARS-CoV-2 Panbio RADT/RT-qPCR tests, which tallied some 5,136 tests (Table 3). Seven hundred and thirty-five samples tested positive by RT-qPCR. Panbio detected 279 (5.4%) positive tested samples. Two hundred and sixty-five samples (5.2%) were assessed as true positive, 14 were false positive (0.3%), 4,387 samples were true negative (85.4%), and 470 were false negative (9.2%). Overall sensitivity (Sn) was 36.1% (95% CI 32.7–39.6%), overall specificity (Sp) was 99.7% (95% CI 99.5–99.8%). PPV was 95.0% (95% CI 91.8–97.0%), and NPV was 90.3% (95% CI 89.5–91.1%).

**Table 3:** Analytic performance of Panbio, all tests included.

	**Panbio positive**	**Panbio negative**	
PCR positive	265 (5.2%)	470 (9.2%)	Sn: 36.1% (95% CI 32.7–39.6%)
PCR negative	14 (0.3%)	4,387 (85.4%)	Sp: 99.7% (95% CI 99.5–99.8%)
	PPV: 95.0% (95% CI 91.8–97.0%)	NPV: 90.3% (95% CI 89.5–91.1%)	

To perform a stratified statistical analysis, 696 parallel samples were excluded to avoid distortion, which were repetitive tests of patients who were aligned to the follow-up of the SARS-CoV-2 RT-qPCR positive cases.

The results are displayed in Table 4. Out of 4,440 paired tests, 609 samples tested positive using RT-qPCR, resulting in a prevalence of 13.7%. Panbio™ detected 251 (5.7%) positive tested samples. In this calculation method, overall sensitivity was 41.2% (95% CI 37.4–45.2%), overall specificity was 99.7% (95% CI 99.4 99.8%). Positive predictive value (PPV) was 95.1% (95% CI 91.8–97.1%), negative predictive value (NPV) was 91.4% (95% CI 90.5–92.2%).

**Table 4:** Analytic performance of Panbio, repetitive tests (696) excluded.

	**Panbio positive**	**Panbio negative**	
PCR positive	251 (5.7%)	358 (8.1%)	Sn: 41.2% (95% CI 37.4–45.2%)
PCR negative	13 (0.3%)	3,818 (86.0%)	Sp: 99.7% (95% CI 99.4–99.8%)
	PPV: 95.1% (95% CI 91.8–97.1%)	NPV: 91.4% (95% CI 90.5–92.2%)	

**Figure 1:**
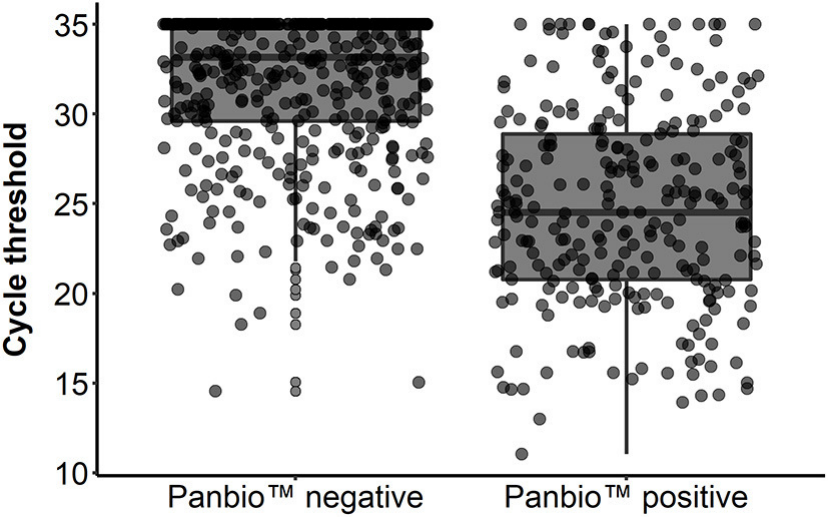
Cycle threshold values in Panbio negative and positive individuals (attached).

Table 5 depicts the sensitivity of Panbio RADT among the different Ct ranges. Sensitivity was 91.2%, in the group of Ct values ≤ 20, 68.6% within the Ct range of 20–25, 47.9% in the group of Ct values between 25 and 30, and 12.6% in the group of Ct values between 30 and 35. The overall mean cycle threshold was 29.2. The median Ct value in Panbio positive individuals (24.5) was significantly lower (*p* < 0.001) compared to the Panbio negative individuals (33.2) (Figure 1 and Table 6). Testing accuracy in the case of symptomatic patients reached the sensitivity of 48.7% (95% CI: 44.4–53.1%) with the specificity of 98.9% (95% CI: 97.9–99.5%). PPV was 96.9% (95% CI: 94–98.4%), NPV was 73.7% (95% CI: 70.9–76.4%).

**Table 5:** Panbio sensitivity among cycle threshold ranges.

**Characteristic**	**Overall, *N* = 735**	**≤ 20, *N* = 57**	**20–25, *N* = 124**	**25–30, *N* = 165**	**30–35, *N* = 389**	***p*-value**
**Panbio results**						<0.001^a^
Negative	470 (63.9%)	5 (8.8%)	39 (31.4%)	86 (52.1%)	340 (87.4%)	
Positive	265 (36.1%)	52 (91.2%)	85 (68.6%)	79 (47.9%)	49 (12.6%)	

a *Pearson's Chi-squared test*.

**Table 6:** Panbio RAD test results.

**Characteristic**	**Overall, *N* = 5,136**	**Negative, *N* = 4,857**	**Positive, *N* = 279**	***p*-value**
**Cycle threshold**	735 (14.3%)			
Mean (SD)	29.2 (5.7)	31.7 (4.1)	24.8 (5.5)	
Median (IQR)	30.6 (25.1, 35.0)	33.2 (29.6, 35.0)	24.5 (20.8, 28.9)	<0.001^a^
Minimum; maximum	11.0; 35.0	14.6; 35.0	11.0; 35.0	

a *Wilcoxon rank sum test*.

### Follow-up of SARS-CoV-2 infections

In the case of those individuals who tested positive on the SARS-CoV-2 RT-qPCR test and were followed up with continuous repeated PCR testing, we observed how many days passed until the first negative RT-qPCR result. We compared the time in days until the first negative RT-qPCR test in the follow-up of SARS-CoV-2 confirmed positive cases. The lowest number of tests per individual was two, and the highest was eleven. There was a significant difference in the median days until a negative PCR test between Panbio negative and positive groups (Table 7).

**Table 7:** Days until negative PCR in comparison with Panbio RAD test results.

**Characteristic**	**Overall, *N* = 291**	**Negative, *N* = 166**	**Positive, *N* = 125**	***p*-value**
**Days until negative PCR**				
Mean (SD)	12 (7)	9 (6)	15 (7)	
Median (IQR)	10 (7, 15)	8 (5, 12)	15 (10, 19)	<0.001^a^
Minimum; Maximum	1; 35	1; 34	2; 35	

a *Wilcoxon rank sum test*.

### Comparison of the general tested population with fatal cases

We compiled demographic and clinical data from 80 individuals who succumbed due to complications related to SARS-CoV-2 and compared it with the generally tested population (Table 8). There was a remarkable difference in gender distribution of the tested cases among the general population who were SARS-CoV-2 suspected, in favor of females: 42.5 vs. 57.5% (male:female ratio). Despite the sex imbalance in the tested population, when comparing the difference of the gender distribution in the general tested population and the population suffering from fatal disease outcome with Pearson's Chi-squared statistics, the gender dominance significantly reflected males, 58.8 vs. 41.2% (*p* = 0.004). The median age difference was also significant (*p* < 0.001): 52 (IQR 30–71) in the general population vs. 78 (IQR 70–87) among the fatal cases.

**Table 8:** Comparison of the general tested population with fatal cases.

**Characteristic**	***N***	**Overall,** ***N* = 4,440**	**General tested population,** ***N* = 4,360**	**Death due to complications of SARS-CoV-2, *N* = 80**	***p*-value^a^**
**Gender**	4,440 (100.0%)				0.004^a^
Male		1,901 (42.8%)	**1,854 (42.5%)**	**47 (58.8%)**	
Female		2,539 (57.2%)	**2,506 (57.5%)**	**33 (41.2%)**	
**Panbio results**	4,440 (100.0%)				<0.001^b^
Negative		4,176 (94.1%)	**4,138 (94.9%)**	**38 (47.5%)**	
Positive		264 (5.9%)	**222 (5.1%)**	**42 (52.5%)**	
**Ct value**	609 (13.7%)				
Mean (SD)		28.8 (5.8)	**29.1 (5.8)**	**27.0 (6.0)**	
Median (IQR)		29.9 (24.5, 35.0)	**30.4 (24.8, 35.0)**	**26.9 (22.8, 32.6)**	0.002^c^
Minimum; Maximum		11.0; 35.0	**11.0; 35.0**	**14.6; 35.0**	
NA		3,831	**3,831**	**0**	
**Symptomatic**	4,440 (100.0%)	1,083 (24.4%)	**1,009 (23.1%)**	**74 (92.5%)**	<0.001^a^
**Age**	4,440 (100.0%)				
Mean (SD)		50 (25)	**50 (25)**	**78 (11)**	
Median (IQR)		53 (30, 72)	**52 (30, 71)**	**78 (70, 87)**	<0.001^c^
Minimum; Maximum		0; 101	**0; 101**	**55; 98**	
**Ct range**	609 (14%)		**529**	**80**	0.019^b^
≤ 20		53 (8.7%)	**44 (8.3%)**	**9 (11.3%)**	
20–25		111 (18.2%)	**92 (17.4%)**	**19 (23.8%)**	
25–30		143 (23.5%)	**118 (22.3%)**	**25 (31.3%)**	
30–35		302 (49.6%)	**275 (52.0%)**	**27 (33.8%)**	
NA		3,831	**3,831**	**0**	

a *Pearson's Chi-squared test.*

b *Fisher's exact test.*

c *Wilcoxon rank sum test.*

*The p-value is referring to the statistical comparison of the highlighted columns in bold face. In categorical variables, p-values are indicated in the row of the characteristic above the group variables they refer to. In continuous variables, the p-values are listed in line of the median (IQR), which is used for the comparison*.

## Discussion

### SARS-CoV-2 RADT performance

Since their first deployment, SARS-CoV-2 rapid antigen tests have undergone several diagnostic accuracy evaluations in a series of large population studies including a nation-wide community-based study (14). The Cochrane library reported an overall RADT sensitivity of 94.5% when reported Ct values were ≤ 25 (95% CI 91.0–96.7%; 36 evaluations; 2,613 cases), meanwhile, Ct values >25 had a sensitivity of 40.7% (95% CI 31.8–50.3%; 36 evaluations including 2,632 cases) (6). Panbio RADT was reported to maintain a high specificity (between 94.9 and 100%) in preliminary clinical studies (15, 16). Krüger et al. (17) demonstrated a sensitivity of 95.8% in Ct values <25 and within seven days from symptom onset. In larger study populations, Panbio sensitivity was between 33.3% (18) and 55.3% (19) in asymptomatic patients. Wagenhäuser et al. described an overall sensitivity of 46.7% (20), meanwhile, Treggiari et al. found the overall sensitivity at 66.8% (21).

Our study began with a review of 5,136 cases in which we found SARS-CoV-2 Panbio RADT overall sensitivity to be low, at 36.1%. Test sensitivity improved to 41.2%, when repetitive follow-up tests were excluded from the analysis, which is primarily due to the exclusion of samples with low viral load close to the maintained cut-off level and the lowest detection limit of the qPCR. The hospitalized population entered our health care system with COVID-19 related, COVID-19 associated and COVID-19 independent problems. The population of which was asymptomatic at the time of the SARS-CoV-2 Panbio RADT and later confirmed to be negative, was relatively high. Using PCR Ct score specific stratification on the data, sensitivity reached 91.2% when the SARS-CoV-2 RT-PCR Ct value was equal or under 20, which corresponds with an extremely high viral load. Sensitivity decreased with the increase of the Ct values: 68.6% in the case of Ct ranges between 20 and 25, 47.9% between 25 and 30, and most of the cases occurred between Ct values of 30 and 35, in which the sensitivity dropped to 12.6%. This sequential sensitivity performance was observed in the aforementioned studies scattered over a wide scale and dependent upon sampling quality and differences of the applied SARS-CoV-2 PCR method. The key factors regarding high sensitivity is seemingly dependent upon the high viral load and presence of symptoms.

According to Platten et al. (22), 52.6% of positive cases with Ct values > 28 were undetected by RAD tests. Our findings are consistent with the above-mentioned study: the 80 patients who succumbed due to complications of SARS-CoV-2 had a median Ct value of 27.0, with a Panbio sensitivity of 47.5%. PPV and NPV are highly dependent upon prevalence. In published literature, PPV with a prevalence <10% was observed between 89.3% (23) and 100% (24). NPV was between 72.2 and 98.3% when prevalence was 6.3% (20). During the study period, the prevalence was reported to be 9.2–14.7% among the Hungarian population (25), in which the epidemic was at the community transmission phase. Overall, PPV was 95.0% (95% CI 91.8–97.0%), overall NPV was 90.3% (95% CI 89.5–91.1%). The PPV and NPV results were influenced when the tested population was stratified according to the presence of symptoms, showing an increase in the case of the symptomatic study group. Results of our clinical study highlight the universal observation associated with SARS-CoV-2 RT-qPCR: it is the most reliable tool in the detection of active SARS-CoV-2 infection. Although SARS-CoV-2 RADT offers several advantages over SARS-CoV-2 RT-qPCR (26), even in clinical settings due to its point-of-care testing (POCT) administration and rapid turnaround time, these tests are less sensitive or at critically low prevalence rate of the infection and can be considered unsatisfactory regarding accurate testing and consequential diagnosis. Our results demonstrate stratification according to symptoms can enhance test accuracy. However, to gain enhanced diagnostic performance, test application recommendations will be needed and adapted to the different phases of the epidemic curve. SARS-CoV-2 RADT is ideally suitable during the exponential and peak plateau phases of an outbreak.

### Follow-up period of positive cases

According to our diagnostic strategy, we can detect SARS-CoV-2 genetic material in positive individuals up to a maximum of 35 days, which did not depend on parallel Panbio positivity. This result confirms PCR positivity alone or a qualitative result from RT-qPCR is not strongly correlated with infectivity regarding the patient. Viral culture studies confirmed SARS-CoV-2 may remain infection competent for 10–14 days following the onset of symptoms (27). Thereby, SARS-CoV-2 RT-qPCR detects remnant viral RNA beyond the time period of recovering replication-competent virus. This aforementioned capability regarding the RT-qPCR is reflected in the current study, which is needed to be taken into consideration when the question of patient admission or discharge arises within a clinical ward. SARS-CoV-2 RADT with corresponding SARS-CoV-2 PCR was found to be a very efficient tool for accurate SARS-CoV-2 diagnostics and estimation of the phase of infectivity.

### Test correlation with disease severity

In the combined SARS-CoV-2 Panbio RADT and RT-qPCR tested population, 80 fatal cases were observed during our study period. Statistical evaluation of the deceased population group identified significant differences compared to the later recovering general population according to gender, age and presence of the symptoms during the first testing and PCR Ct stratification distribution. Older age, male sex, clinically symptomatic status and lower Ct range are all significantly correlated to disease fatality. However, we emphasize, six of the patients (7.5%) had no clinical symptoms during the first test and SARS-CoV-2 Panbio RADT was negative in 38 individuals, 47.5% of the fatal cases. These numbers suggest rapid antigen testing should not be the sole test administered to populations at high risk of developing severe disease.

## Conclusions

Our study involved the highest population size related to SARS-CoV-2 Panbio RADT and we could follow-up the SARS-CoV-2 Panbio RADT and RT-qPCR positive population with PCR testing until recovery or the mortality outcome. Our study draws attention to strengths and weaknesses regarding the RAD testing in clinical applications. We recommend the SARS-CoV-2 Panbio RAD tests be used as the sole testing modality only among high population prevalence rates of the infection and to be used for symptomatic individuals with average or low severe disease development risk. In the case of high risk regarding the development of severe infection complications, parallel SARS-CoV-2 RT-qPCR consistently needs to be carried out to assure proper diagnostic accuracy and avoid delaying appropriate medical care.

## Limitation

No information was known regarding the onset of symptoms, which should be taken into consideration when comparing it with diagnostic performance. There was no possibility to repeat the RAD or RT-qPCR tests from the same samples due to continuous high daily activity, which leaves open the possibility of human error (e.g., RAD test evaluation beyond the recommended timeframe), despite being performed and evaluated strictly by healthcare professionals. In Hungary, the dominant genetic variant of SARS-CoV-2 was the B.1.1.7 (Alpha) variant during the study period (between 21 January and 30 April. 2021). Although in laboratory-based investigations referencing the performance of the Abbott- Panbio RAD was not affected by the variants, the COVID-19 Antigen study (COVAG) in a real-world setting including 2,215 participants and 338 rRT-PCR confirmed SARS-CoV-2 positive cases Abbott-Panbio RAD test performed 72.3% sensitivity in carriers of the Alpha variant, compared to 84.0% in cases infected with wild-type SARS-CoV-2. The test-sensitivity diminishing effect of the Alpha variant was also observed in the Roche-RAD test (28). Although it is the only study indicating the lower sensitivity regarding the Abbott-Panbio RAD test for the Alpha variant, its authors cannot explain this finding and it must be taken into consideration regarding the relatively low sensitivity and positive predictive value in our results.

## Data availability statement

The raw data supporting the conclusions of this article will be made available by the authors, without undue reservation.

## Ethics statement

The studies involving human participants were reviewed and approved by Regional Committee for the Research Ethics at University of Pécs Clinical Center, reference number 8668-PTE 2021. Written informed consent to participate in this study was provided by the participants' legal guardian/next of kin.

## Author contributions

ÁH drafted the original manuscript. ÁH and KFi participated in data collection. AV was responsible for the statistical analysis. RV-R, HG, KFu, and PU performed the RT-qPCR tests. ÁH and KG conceptualized and edited the manuscript. GK made the final corrections. All authors agree to be accountable for the content of the work and contributed to the article and approved the submitted version.

## Funding

Project No. TKP2021-EGA-13 has been implemented with the support provided from the National Research, Development and Innovation Fund of Hungary, financed under the TKP2021-EGA funding scheme.

## Conflict of interest

The authors declare that the research was conducted in the absence of any commercial or financial relationships that could be construed as a potential conflict of interest.

## Publisher's note

All claims expressed in this article are solely those of the authors and do not necessarily represent those of their affiliated organizations, or those of the publisher, the editors and the reviewers. Any product that may be evaluated in this article, or claim that may be made by its manufacturer, is not guaranteed or endorsed by the publisher.
